# The Hospital. Nursing Mirror

**Published:** 1898-08-20

**Authors:** 


### "The Hospital" Nursing Mirror.

Being the Nursing Section of "The Hospital."

[Contributions for this Section of "The Hospital" should be addressed to the Editor, The Hospital, 28 & 29, Southampton Street, Strand, London, W.C., and should have the word "Nursing" plainly written in left-hand top corner of the envelope.]

### News from the Nursing World.

### THE GREEK PRINCESS AND THE HOSPITALS.

One good thing has been accomplished by the unfortunate Turco-Greek war—the Crown Princess of Greece has turned her attention to nursing. Although the war is over she is still engaged studying, founding and organising hospitals, and all that appertains thereto. During her visit to England she visited as many institutions as possible, and gave Netley a surprise visit, which was most successful. Her Royal Highness, who was accompanied by the Duke of Sparta and the Princess Victoria of Schleswig-Holstein, inspected a number of wards occupied by men wounded in the India frontier war.

### ROYALTY AT ABERDEEN.

A bazaar is to be held at Aberdeen on October 28th in aid of the Royal Hospital for Sick Children. If enough money is raised for the purpose it is intended to enlarge the patient's accommodation, and to build and furnish a new home for the nurses. H.R.H. Princess Henry of Battenberg has promised to open it.

### DISTRICT NURSING IN ARGYLLSHIRE.

Her Majesty expressed her sympathy with the sick poor by founding the Jubilee Nursing Institute. Her wish that all should enjoy skilled nursing in sickness has taken root in the hearts of her subjects with remarkable kindliness. The scattered hamlets, as well as the thronging towns of Scotland, are either blessed, or to be blessed, with the work of the trained district nurse. Last year the Duke of Argyll put himself at the head of a movement to celebrate the sixtieth anniversary of Her Majesty's accession by founding a nursing association for the county of Argyll, and the sum collected for this object reached £1,230. The association began work a year ago with four nurses, and soon eight nurses will be at work. This year the Duchess is working to ensure the continuance and extension of the same society already successfully launched, and with this intent addressed a large meeting in the town hall, Campbeltown, on the 2nd inst. called for the purpose of preparing a grand bazaar to be held at Glasgow in November next. The bazaar promises to be a successful one. The Queen is one of the patrons. Sir Thomas Lipton is to be present, and if the Duchess brings a few more purchasers of the same stamp as the gentleman who could afford a cheque of £25,000 for the Princess of Wales's dinner party on Jubilee day, the funds of the society will no doubt reach a very satisfactory figure.

### A MONTHLY NURSE'S FEES.

A case of considerable importance to monthly nurses was heard on the 3rd inst. before Judge Sir W. L. Selfe. Mrs. Rice engaged Mrs. Hartly to nurse her from May 15th. Mr. Rice afterwards retained her services from April 6th to May 15th at half fees, and the confinement did not take place until June 11th. Mr. Rice, a month from the last named date, gave Mrs. Hartly a cheque for £7 17s. 6d., who declared her intention of suing him for another £5 5s.; that is, she claimed as her just debt £2 12s. 6d. retaining fee from April 6th to May 15th; £5 5s. from May 15th to June 11th; and £5 5s. from June 11th to the date of her leaving the case. The Judge held with the plaintiff, and gave judgment for the amount claimed. The interest of this case seems to lie chiefly in two facts; first, that it was given in the nurse's favour, even although the agreement was not in writing; and, secondly, that the Judge held the defendant liable for the full amount of the fee during the time that the nurse was waiting for the confinement to come on. We would, however, warn nurses not to depend too much upon this decision. The question of a written agreement hardly arose, for the facts were practically undisputed. No one, therefore, can properly argue from this case that a written agreement is unnecessary. The Judge, in summing up, is reported to have said "it appeared to him that there must be some date fixed for the engagement of a nurse, and if the calculation turned out to be erroneous it appeared only reasonable that the nurse should be paid for the time she had been retained. Mr. Rice had said that had the plaintiff gone a week after the confinement he would have expected to pay her 7½ guineas for the time she had been waiting, and that being so, it appeared to him that it put an end to the case. Plaintiff had not only waited up till that time, but she had rendered her services for nearly a month afterwards. There would be judgment for plaintiff for the amount claimed."

### THE SUPPLY OF INFIRMARY NURSES.

Mr. Chaplin's reply to Mr. Talbot in the House of Commons on August 4th on the subject of training nurses for the workhouse infirmaries shows how little hope there is of the Local Government Board lending a helping hand to the Guardians in the dilemma in which the order of August, 1897, has placed them. It may be remembered that one of the chief reasons given by the Workhouse Nursing Association for relinquishing the work of training nurses for poor-law institutions was that the association could not hope to meet the increased demand for nurses resulting from the order, and that the work it was doing should be carried on by a larger and State-supported society. Of course, many people expected that the Local Government Board would organise or help such a society, but beyond promising to encourage the training of nurses the Board will do nothing. Mr. Chaplin's reply in full is as follows: "The order of August, 1897, provides that no person shall be appointed to the office of nurse without having had such practical experience of nursing as may render her a fit and proper person to hold the office. Since the issue of that order there has been a decided improvement in the nursing arrangements in many of the workhouses, and of the nurses appointed an increasing number have been trained. I am aware that, in consequence of the great demand for fully-trained nurses, many Boards of Guardians have found difficulty in obtaining the services of such nurses. The Local Government Board, however, have neither the duty nor the power to supply nurses for workhouses; but they are encouraging the training of probationer nurses at workhouses where adequate arrangements exist, who will increase the available supply, we hope, in the future."

### INSPECTION SERVICE.

The Government of India has sanctioned a grant of five rupees a day to the lady superintendents of the Indian Nursing Service when they are absent from their stations on inspection tours. This grant is in lieu of the present detention allowance.

### THE VICTORIA COTTAGE INSTITUTE, CAWTHORNE.

The Victoria Cottage Institute is Colonel and Mrs. Montague Spencer Stanhope's Diamond Jubilee memorial gift to the little village of Cawthorne, near Barnsley. Colonel Stanhope found the money to fit up a disused Wesleyan chapel as a nurse's residence and gathering-place for the parish, and Mrs. Stanhope thought out the plans and was the moving spirit in carrying them out. Dr. Eden, Bishop of Wakefield, performed the opening ceremony, at which there was a large attendance, on the 9th inst. The new institute is under the management of a committee of ladies, who have engaged Miss Nightingale as district nurse. She and a caretaker reside at the institute, and a large room has been added, where girls are to be taught cookery, and which will be used for mothers' meetings. The scheme therefore promises to develop into a centre of useful parish work. Apart from the success of this individual instance, there is much to commend the idea of co-operation in parish philanthropy. Cheapness has been sought in village communities by employing the cottage nurse of inferior training, but those who are experienced in the working of this plan acknowledge —some very reluctantly, it is true—that where the money can be raised the fully-trained nurse is better, and often cheaper. Co-operation affords opportunities of economy that cannot be attained by any other means, and a district nurse will not find her usefulness hampered by seeing her patients occasionally when they come to a meeting or a cookery class, or perhaps to a social entertainment held at the class-room attached to her residence.

### HOLIDAY MAKING.

The nurses of the North Stafford Infirmary, Stoke-upon-Trent, have enjoyed two delightful holidays this season through the kindness and generosity of the honorary surgeons connected with the institution. Mr. W. D. Spanton invited them in two detachments to a garden party at his house, which is prettily situated in the country, and where they spent a very pleasant time. Mr. and Mrs. G. H. Hatton also arranged a couple of picnics for them; one half of the nurses going to the first, and the others to the second. On each occasion a saloon carriage conveyed the guests from Trent Station, Stoke, to Chester, where, after seeing the cathedral and other places of interest, they had an excellent luncheon at a restaurant. They then went up the river Dee (on a steamboat, chartered for the occasion) as far as Eton Hall. Here there is a charge of sixpence a head for admission, which goes to the funds of the Chester Hospital, The Duke of Westminster's gardens were very beautiful, the weather perfect, the arrangements good, their hosts (who bore all expenses) most kind, and the holidaymakers returned to Chester to a liberal tea, feeling that nothing more was needed to complete their pleasure. The nurses have all thoroughly appreciated it, the more especially as this is the first occasion on which such a treat has been given them.

### THE KLONDYKE CONTINGENT.

There is a rumour afloat to the effect that the Klondyke contingent of the Victorian Order of Nurses are not altogether a success. The various reports on other subjects that have hailed from this golden Eldorado have been bewildering, for the one not infrequently flatly contradicted that preceding it. It would be unwise, therefore, to put too great credence to any but those given authenticated authority, and even then the multitudinous incidents, adventures and predicaments with which the nurses have been confronted, test the powers of resource and adaptability of the woman rather than her skill as a nurse. If the report which we hear speaks true the ladies prefer to work as doctors rather than as nurses, and as their knowledge of the medicine is not equal to the demands made on it failure is inevitable. Fully-trained medical women have obtained recognition from the learned professions and from the public; but the same bodies that wage deadly war on quacks will not be disposed to tolerate them when masquerading in a nurse's uniform.

### THE VICTORIA NURSING INSTITUTION, WALSALL.

The Jubilee Memorial of Walsall is now fairly established. From the 1st inst. trained nurses will be available for the service of the sick poor. Some little time ago we chronicled the fact that No. 40, Bradford Street, had been taken as a nurses' home, and that Miss Eyre had been appointed lady superintendent. An executive committee of six ladies and six gentlemen has been appointed, and cases recommended by any clergyman, minister, or medical man will be nursed free of charge. The institution has trust funds to the amount of £2,500, which will yield an income of £70 a year, but the expenses of the home are expected to average £200. As there is great need of the nurses, it is to be hoped that the public will support the new charity liberally.

### SHORT ITEMS.

Plague is still present in the Rangoon municipality, and an additional nurse has been sent out from home to attend to cases of emergency. Another nurse has also gone to Rangoon from India to take special duty for the Port Trust.—It has been decided to authorise the master of the Driffield Workhouse to engage temporary nurses from time to time as required. This step is taken in consequence of the action of the Local Government Board pointing out that the nursing staff under the present arrangements is inadequate. The third annual meeting of the St. Agnes District Nursing Association was held lately, and a prosperous year's work was recorded. The South African emigrants have again subscribed liberally.—Twenty of the nurses belonging to Hucknall Torkard District Nursing Association were entertained at Kingston by Lady Belper, the president of the Notts District Nursing Association. The guests enjoyed themselves well.—Major-General Hallowes, who is commanding the troops in Jamaica, is endeavouring to raise money to provide two trained nurses for the military hospital there, the dearth of trained nurses having been severely felt during the late outbreak of yellow fever.

### Antiseptics for Nurses.

By a Medical Woman.

### XIV. — COMPOUNDS OF FORMALDEHYDE—ALCOHOL, &c.

One of the best known of the formaldehyde compounds goes by the name of glutol. It is composed of gelatine and formalin gas as follows: A warm aqueous solution of gelatine is prepared, and to this added formalin gas in such proportion as to make it a two per cent. solution. The mixture is then very thoroughly stirred together so as to secure a complete combination, and thus is formed a new chemical compound possessing entirely different properties, as the formalin has been chemically combined in the mass, and not merely mixed with it. When the mixture has become cold the gelatine is found to have completely lost its original gelatinous properties, and has become a hard transparent resistant mass, having a glassy lustre; it can be pounded up into a fine powder, and in this form is used as an antiseptic dressing in surgery. It is described as being an inactive stable compound, under ordinary conditions, but is quite insoluble in water, in dry or moist heat, in all acids and alkalies; when heated, however, it becomes slightly extensible; but regains its former stiff elastic nature when it becomes cold again. When it comes into contact with animal tissues and secretions it becomes gradually decomposed by their action, and thus formalin vapour is set free, and acts continuously as a very satisfactory and effective antiseptic in the opinion of those continental doctors who have used glutol. If the decomposition of the glutol and the consequent liberation of formalin vapour is found to be too slow, however, as is sometimes the case in such wounds as varicose ulcers and gangrenous wounds generally, where dried secretion and necrotic tissues, &c., prevent the antiseptic from coming into immediate contact with the healthier tissues beneath, under such circumstances it has been found by Dr. Schleich, of Berlin, that the use of a few drops of a mixture of pepsin 75 grains, hydrochloric acid 5 minims, and distilled water to 4 ounces, poured over the layer of powdered glutol after it has been sprinkled over the wounded surface will cause the gelatine to dissolve more rapidly, and will consequently set free the formalin vapour more readily. The advocates of glutol contend that the constant development of formalin vapour beneath the scab formed by the powdered glutol keeps up a continuous antiseptic action, and thus, according to Dr. Schleich, is avoided one great objection to the use of ordinary antiseptics, with which a temporary and for the time very energetic contact action of the antiseptic may take place, but in consequence of the formation of a quantity of almost insoluble compounds between the antiseptic and the healthy tissues there is a complete arrest of all subsequent antiseptic action until the wound has been cleansed and fresh antiseptic applied. This is not the case with glutol, which forms a firm impermeable scab when in contact with clean antiseptic wounds after a few hours, and renders all other disinfectant measures unnecessary, as the scab is both aseptic and protective, and the wound heals very satisfactorily beneath it and without acquiring renewal of the glutol. Glutol has been found specially useful for wounds n parts where it is difficult to apply a bandage, and where it causes inconvenience to the patient. It has also been used with very marked success by Thomalla in the treatment of burns. As regards the uses to which glutol can be put, Dr. Schleich says: The complete replacement of glutol with connective tissue by means of cell activity points to a further clinical employment of this material. As it can be poured out and dried in moulds of any desired shape, and when heated can also be bent as desired, it appears to be eminently adapted for plastic surgery in supplying defects of all kinds. Moreover, when impregnated with calcium salts, as suggested by A. Gottstein, it forms hard connective tissue of any desired forms which attaches itself to the bony matrix, and can be used to form artificial bones instead of resected pieces of bone.

Glutol is also prepared in what are called glutol skins, that is, the gelatine and formalin gas mixture is spread out very thin and allowed to dry as a film or skin. These films are very useful in superficial wounds or merely abrased surfaces, when a protective covering like plaster is desirable. The film merely requires warming to make it perfectly pliable, and is then applied and kept in place with a turn or two of bandage until it adheres to the wound, which must, of course, have been previously rendered perfectly aseptic.

Steriform is a combination of lactose and formaldehyde, and is also in the form of a powder.

Formo-gelatine is the name of a similar compound of formaldehyde and gelatine, and is apparently identical in all respects with glutol. It is a grey, somewhat gritty, mobile, and odourless powder, and is a good substitute for iodoform, but possessing all the powerfully antiseptic and non-toxic properties of formaldehyde.

The use of absolute alcohol as a disinfectant for hands and instruments has quite recently been revived in America. Absolute alcohol has long been known as an excellent antiseptic, but of late years newer antiseptics have superseded it. An American surgeon has however lately proved by experiment that absolute alcohol does not impair the edges of cutting instruments as is the case when they are boiled in normal salt solution, which decidedly dulls their edges. The same authority also declares it the quickest disinfectant that can be relied upon for disinfecting the hands, and that scrubbing them in 90 per cent. alcohol for five minutes takes up both the fatty matters of the skin and also the bacteria, which are thus washed away. From the experiments, however, it would appear that instruments can be thoroughly disinfected in alcohol only so long as they have not been infected with pyogenic organisms, but have only been infected under the conditions which ordinarily surround us in everyday life. Consequently the use of absolute alcohol as a means of disinfecting instruments is of necessity very limited.

Enterol is a watery, transparent liquid, which is apt to turn brown when exposed for long to the air. It has a taste and smell resembling those of kresol. It is slightly soluble in 100 parts of water. It is said to possess about six times the strength as an antiseptic of carbolic acid, to which it is very similar in properties. When diluted to a strength of 1 part in 500 of water it has been used as an internal antiseptic.

Microbene is described as a non-poisonous, stainless, non-corrosive disinfectant, and is a product manufactured from the antiseptic and microbicidal constituents of coal tar. It is claimed that it is the strongest non-poisonous disinfectant in existence. Microbene is a thick, dark-coloured liquid with the characteristic smell of coal tar. When diluted with water to 100 times its volume it forms a milky fluid, having an alkaline reaction, and in this form it is recommended for disinfecting purposes. Microbene contains a moderate amount of the cresols which render it an efficient disinfectant. The form in which it is supplied, viz., quarter-pound tins, suffice to make 25 pints of disinfectant.

Bensolyptus is an agreeable and effective combination of various pleasant antiseptic substances. It is a clear, colourless liquid, possessing the delicate aromatic smell of gaultheria. It contains, amongst others, boric and benzoic acids, which give it its chief claim to being an antiseptic. It is a mild, non-irritating antiseptic, chiefly useful as a mouth-wash, &c.

### A Book and its Story.

### A TALE OF MAGIC: "DR. KOOMADHI OF ASHANTEE."

MR. FRANKFORT MOORE has fully established himself as a popular writer of romance, and his latest contribution to literature, "Dr. Koomadhi of Ashantee,"* will be hailed by many a reader who requires a bright little story to beguile their holiday leisure. The *mise-en-scene* of this tale of magic is laid at Picotee, a certain malarial town situated on the West Coast of Africa. The story opens with a description of the distant township as seen from the s.s. "Penguin."

To the eyes of all aboard the mail steamer "Penguin," "which has just run up a Blue Peter in the anchorage, the town seemed of dazzling whiteness. It was only the inhabitants of Picotee who knew that the walls of the houses were not white, but of a sickly yellow tinge; consequently it was only the inhabitants who knew how inappropriate it was to allude to their town as the 'whitened sepulchre,' a term of reproach which was frequently levelled against it rather on account of the appalling percentage of the mortality among its inhabitants than by reason of the spotlessness of the walls, though they did appear spotless when viewed from the sea."

The thermometer registered 95 deg. in the saloon of the "Penguin." . . . Dr. Claude Koomadhi, who occupied a villa built on the lovely green slope above the town, "opened the shutters of the room in which he sat, and listened to the song of the pepper bird." Upon his features, which seemed as if carved out of black oak and delicately polished, a sentimental expression appeared. His eyes showed a large proportion of white as he sighed, and remarked to his servant that the song of the pepper-bird reminded him of home.

"Of 'ome, sah?" said the old woman. "Lor bless yah, sah, dere ain't no peppah-buds at Ashantee." Dr. Koomadhi's eyes no longer wore a sentimental expression. "You don't understand, Sally; I said home—England," remarked the doctor.

"Oh, beg parding, sah; thought you 'looded to Ashantee," said the old woman, as she rolled out of the room, still uttering a senseless laugh.

Dr. Koomadhi did not seem to be greatly put out by the reminder of the fact that Ashantee was his birthplace. He threw himself back in the chair and took a sip from the tumbler, and resumed his perusal of the *Saturday Review*, brought by the "Penguin" in the morning. Next, Dr. Koomadhi gave himself up to a reverie of his past and future life. In the past all the obstacles which had been in his path to success he had conquered with commendable pertinacity, and from these and such like premises the doctor now argued that there was no reason why he might not overthrow in the near future all that might threaten to bar his progress—and yet he was uneasy. "He did not become more settled when he had gone to a drawer in his writing-desk and had taken out a cabinet portrait—the portrait of a lady, and had gazed at it for several minutes." He walked fitfully up and down the room for another hour. "Then he opened his shutters, and the first breath of the evening breeze came from the sea upon his face." "I'll do it," he said, resolutely. "Why should I not do it? Surely that old ridiculous prejudice is worn out. Surely she at least will be superior to such prejudices. Yes, she must——"

His mind made up, elated with his success in the past, Dr. Koomadhi, with a commendable hopefulness, gives himself up to a yet greater prospect for the future, and approaches the Residency to lay his heart and his fortune at the feet of Commander Hope's daughter—Commander Hope being the Commissioner of Picotee. After certain preliminaries duly described by Mr. Moore, the negro doctor epitomised his proposal in the following manner :—

"The future is in your hands, Miss Hope. I have come here to-day to tell you that I have never loved anyone in all my life but you, and to ask you if you will marry me." There was a long pause . . . and at the same time a baboon came in front of the window and raised his right hand to the salute.

"You are mad—mad!" she said in a whisper that had something fierce about it. Then she lay back in the chair with a laugh. "I marry you. I should as soon marry—." She had pointed to the baboon before she checked herself.

"You would as soon marry the baboon as me?" said he in a low and laboured voice.

More followed, and then the disconsolate suitor retired from Miss Hope's presence, only to learn of the existence of a more fortunate rival in the field, and that the Commissioner's daughter had plighted her troth to another.

The marriage of Major Minton and Miss Hope took place shortly after this. To mark the happy event there was a good deal of unofficial and wholly irregular burning of gunpowder. Dr. Koomadhi spent several hours of the afternoon amputating fingers of Krooboys that had been mutilated through an imperfect acquaintance on the part of the native populace with the properties of gunpowder when ignited. An eye or two were reported to be missing, and in the cool of the evening the doctor had brought to him, by a conscientious townsman, a human ear for which no owner could be found.

Mrs. Minton was married and overwhelmingly happy, and "Dr. Koomadhi was showing day by day that he had forgiven her," and "thus it was felt that he was worthy to be regarded by all men as a gentleman and a Christian." It was not everyone, however, who thought so leniently of the nigger doctor. The Commissioner's secretary, for instance, was given to an insistent reiteration that the veneer of civilisation was but a veneer after all, and that the nigger born was a nigger to the end of his days. It is from this point onwards that the dramatic element in Mr. Frankfort Moore's story develops, and the supernatural is introduced in an effective manner through the intervention of the agency of a certain miraculous "stone" in the possession of Dr. Koomadhi, which the Doctor uses in a manner adverse to the comfort of the fortunate possessor of Miss Hope's hand and heart.

To this end the magic of the "Kabela" and of the "Sacred Ear" are brought into play by the doctor, who possesses himself of both, and works havoc on his victim. The accounts of the transformation effected on Major Minton are highly imaginative and amusing—to the reader—though of a more tragic nature to those affected, but the owner of the magic stones continues with unrelenting vengeance to wreak his pique on the husband of the girl who had insulted him with her refusal of marriage. For the details of Major Minton's strange career whilst under the influence of the charm we must refer the reader to the author's own account, which cannot fail to amuse.

The story winds up in a happy manner. When the book closes we have husband and wife restored to blissful married life, whilst he who intervened with his direful magic becomes himself a victim, and his end is a tragedy where punishment is meted him in proportion to his deserts.

* "Dr. Koomadhi, of Ashantee." By F. Frankfort Moore. (London: Archibald Constable. 1898.)

### Wants and Workers.

HOME FOR GENTLEMAN.—Can you tell me of any home or private house in which a gentleman could be accommodated? He requires the attention of a nurse occasionally. The south coast of England, Southsea preferred; or Harrogate. No medical attendance is required. —J. B. W.

[Can any of our readers personally recommend such a home?]

### Training in the Provinces.

(*Continued from page* 165.)

THE CUMBERLAND INFIRMARY, CARLISLE (103 Beds).

Terms of Training.

All probationers are received at the Cumberland Infirmary on the same terms. The period of training is for three years, but nurses are bound by a four years' agreement, serving during the fourth year as charge nurse, or on the private staff. A certificate is given at the end of the third year after examination. Candidates should be between 23 and 35 years of age. An entrance fee of £1 1s. is required, and further fees for instruction — first year, £1 1s.; second year, £1 1s.; third year, £2 2s. By the form of agreement signed by the probationer a forfeiture of £20 is required should she break her engagement.

During the first year no salary is given; during the second year salary begins at £15, rising for the third year to £18, and for the fourth to £25. Indoor uniform is provided by the hospital; the wearing of outdoor uniform is optional.

One special advantage is offered to nurses training at the Cumberland Infirmary, a course of three months' training in fever nursing being arranged for during the third year.

The senior probationers take night duty for three or four months at a time under the supervision of a night nurse.

Hours On and Off Duty.

Probationers and staff nurses' hours on duty are from 7 a.m. to 8.30 p.m. on three days in the week, with three hours off in the afternoon, and time for meals. On alternate days they are on duty from 7 a.m. to 7 p.m., with time for meals, taking their off duty time from 7 p.m. to 8.45 p.m. On Sundays they are off duty either in the morning or the evening. No regular "days off" are given, but an occasional half day as can be arranged. The afternoon off-duty hours are from 2.30 to 5.30.

Meals.

Probationers and staff nurses breakfast at 6.40 a.m., lunch is in the dining-room from 10 to 11 a.m., dinners are at 1.30 and 2 p.m., tea at 5 and 5.30 p.m., and supper is at 8.45 p.m. All meals are served in the dining-room, and there are no food allowances.

DERBYSHIRE ROYAL INFIRMARY (185 Beds).

Terms of Training.

Candidates for training at the Derbyshire Royal Infirmary should be not under 21 nor over 32 years of age. They are required to engage to serve the infirmary as probationers for a term of three years from date of appointment, at the end of which certificates are granted to those who have passed satisfactorily through their training. They do not pay any premium or entrance fee, receiving no payment for the first year and commencing at a salary of £10 the second year. For the third year this is raised to £16. Sisters' salaries range from £30 to £36. Probationers in their first year provide their own uniform, afterwards all necessary indoor uniform is supplied to nurses by the hospital, and the wearing of outdoor uniform is optional, but if worn must be of the regulation pattern approved by the Nursing Committee.

A trial of two months is required before a probationer is appointed, and if after appointment she desires to break her engagement she forfeits the sum of £5 and must give three months' notice in writing. If at the end of the first year a probationer has not given satisfaction the infirmary authorities are at liberty to dismiss her.

Three years' training is obligatory for promotion to the post of ward sister, such appointments being made solely upon the ground of special fitness for any position vacant. There are special terms for a limited number of paying probationers. Lectures are given to the nurses by members of the honorary medical staff and by the matron, and examinations held.

Hours of Work and Times Off Duty.

Probationers and staff nurses' hours in the wards are from 7 a.m. to 9 p.m., deducting time for meals and two and a half hours' daily recreation. Sisters are on duty between 8 a.m. and 9 p.m., with two hours off duty daily. Once a week they have a "long evening," from 4.30 to 10 p.m.; a day and a night off once a month, from 7 p.m. one day to 9 p.m. the next; and four hours on Sunday. Staff nurses and probationers also have a day and a night off once a month and four hours' leave on Sundays, from 9 a.m. to 1 p.m. one Sunday and from 5 to 9 p.m. another. Two weeks' holiday is given during the year to first and second year probationers, and three weeks to third year probationers and to sisters.

Night nurses are on duty from 8.55 p.m. to 8 a.m., and after their dinner, at 8.30 or 9 a.m., are off duty till noon, when they are expected to be in their rooms.

Meals.

The time table for nurses' meals is as follows: Probationers and staff nurses' breakfast is at 6.40 a.m., lunch at 9 to 9.30 a.m., dinner at 1.30 p.m., and supper at 9.10 p.m. Sisters breakfast at 7.45 a.m.; dinner is at 1 p.m.; tea at 4, and supper at 8 p.m.

All meals are served in the nurses' dining-room, except the midnight meal of the night nurses. The sisters have their tea in their own sitting-room in the nurses' home.

### The Soldier's Nurse.

The *Regiment* has brought to notice quite a charming little story of the Princess Christian and one of her soldier sons when a small boy, still in the nursery at Cumberland Lodge. Walking with the Princess one day they happened upon some cavalry exercising in the park. "I should like to be a soldier," said the small Prince." "But, my dear," remarked his mother, "you cannot be." "Why not, mamma!" persisted the little fellow. "Oh," said the Princess, posed for the moment, as so many of us are by children's questions, "you would have to leave us; you would have to give up nurse, and you wouldn't like that." The little Prince thought a moment, then he spied a weak point in the maternal logic. "I need not give up nurse, mamma," said he. "But soldiers don't have nurses, dear," replied the Princess. "Oh, yes," retorted the small boy, confidently; "whenever we drive down the long walk on Sunday we see lots of soldiers, and they nearly always have nurses with them."

### Appointments.

MATRONS.

Pembrokeshire and Haverfordwest Infirmary.—On August 12th Miss M. Barwick was appointed Matron here. She was trained at the General Infirmary, Leeds. Her previous appointments were as follows: Sister in charge of 54 beds and of the operating theatre at the Salop Infirmary, Shrewsbury; night superintendent at the Royal Infirmary, Aberdeen; matron of Kincarrathic Private Hospital for the Insane, Perth; matron of the Cottage Hospital, Bridgend; and matron of the Llanelly Hospital.

Wirral Hospital and Dispensary for Sick Children, Birkenhead.—Miss Emma Smith, sister of this hospital, has recently been appointed Matron. She was trained at St. Saviour's Infirmary, East Dulwich Grove, where she also became sister.—Miss Constance Winifred Jones, who was also trained and afterwards sister at St. Saviour's Infirmary, has been appointed Sister at the Wirral Hospital.

Westminster Hospital. — On August 9th Miss Mabel Helen Cave was appointed Matron of this hospital. She was trained at the London, where she finally held the position of assistant matron. Since May, 1897, she was matron of the Metropolitan Hospital, Kingsland Road.

### Some Qualifications of a Really Good Nurse.

NURSING is a high and noble calling, and is rendered no less so by the responsibilities and labour involved. We are thankful to say that those associated with what was once known as "the fashionable nursing craze" are now in the minority. The truehearted woman shrinks from adopting nursing merely as a means of gaining popularity. Many do admittedly find in it their only means of support, and to this there can be no objection, but unless it is chosen from a high principled standpoint, having love as its mainspring, it will not only prove a failure so far as the nurses' feelings are concerned, but may, indeed, have unpleasant results. Leaving the comforts, nay, even the luxuries of home, probationers enter the hospital there to undergo training to fit them to wait upon the sick and suffering.

But while admitting that training is of paramount importance, the question may well be asked, Is it all that is requisite for a nurse? We think not. One may have a good understanding of medical and surgical work, and yet come far short of what is implied in the term "a good nurse." The reason is not far to seek. Training, in the ordinary acceptance of the terms, makes the nurse theoretically, and in a measure practically, acquainted with the various duties which devolve upon her according to the nature of each case placed under her charge, but it can go no further.

This mental and practical training may, and as a matter of fact must, develop the mind and widen the experience of the nurse, but it cannot change her nature. Her natural disposition remains the same, and as this will play an important part in the course of her nursing career it calls for special attention. For one might say that as in the world of thought there are "many men, many minds" so in a hospital ward there are many patients, many dispositions, scarcely two alike. One may be patient in the midst of sickness, yea even in agony; another may be fretful although suffering only slightly. Here then is the place where the character and capabilities of the nurse are tested. If love has been the mainspring of her choice she will bravely face her duties with steadfastness of purpose. Clearheaded and firm, yet gentle withal, nothing will be beneath her notice; every duty will be performed with great exactitude, her responsibilities both to God and man never being lost sight of. In this way patients will soon learn to place in her that confidence which is begotten by love. She will cast an air of sunshine as she moves from one bed to another, attending to their varied needs and speaking sympathetically and encouragingly those kind words so cheering to the sick and suffering.

But here a word of caution—and this leads us to consider another essential quality—sympathy and tenderness must never be regarded as synonymous with familiarity of manner. The nurse possesses, by virtue of her position as such, a dignity which must never be bartered. Not that she should seek to assume an air of superiority, acting as if the patients were beneath her—far from it; but at the same time it is quite possible for a nurse to be gentle and sympathetic with her patients, entering into their troubles in a manner which shows she is interested in their welfare, yet allowing no familiarity. In the ward of such a nurse there will be no gossip.

The disease of one patient should not be discussed with another; to do so marks the nurse as lacking in one of the first qualifications—good judgment. Indeed, unless she has permission to do so (and that is highly improbable) she should not discuss his or her own illness with the patient. If the exigencies of the case require that the patient should know the worst it usually falls to the nurse's superiors to "break the news," and is only committed to her under some exceptional circumstances. Consequently she should maintain the utmost reticence regarding the serious condition of a patient. This applies even more strongly to surgical than to medical cases. For where a patient has undergone an operation, and may be lying in a more or less critical state, unguarded speech as to patients' danger may have very dire results. The non-success of previous similar cases should on no account be repeated to, or in the hearing of, a patient—the successes may. In the same way the affairs of the institution in which she serves, as well as those of her superiors, so far as known to her, should be held sacred. If she speak of her superiors at all, it should be with the respect due to them.

Another very important feature in a good nurse is the tone in which she addresses her patients or converses with others while in the ward, and the demeanour with which she acts in cases of emergency. Her voice should never be highly pitched, nor should she on any account get excited or flurried. In the ward a nurse should possess great self-command. Extreme cases may occur in which she will require to use unusual firmness, but these will be so exceptional as to but prove the rule here laid down. Sick people generally, and those suffering acute pain in particular, are very easily disturbed by noise or excitement; and any unusual commotion may prove at once a deterrent to the convalescent and disastrous to those in danger.

To sum up, then, we would say that a thorough knowledge of her work, combined with high principles, the outcome of which will be strict attention to duty, singleness of aim, a calm, firm demeanour, and a cheerful disposition are the chief qualifications of a really good nurse. META.

### Death in Our Ranks.

IT is with deep and sincere regret that we are compelled to announce the death of Miss Lucy Haward, sister-in-charge of the Ripon Hospital, Simla, which took place on the 14th inst. Although the deceased lady had not been in robust health for some time past, yet she had been actively engaged in her professional duties, and no suspicion had been entertained of her health being seriously affected, still less of any fatal issue. She had, however, been suffering from a slight degree of cardiac weakness; it was also known she had taken greatly to heart the death of her sister, which occurred recently. Insomnia had supervened, and exerted in her case its baneful and depressing effects, so that she became unable to cast off the weight and anxieties attendant on her daily work as she had done in her more buoyant days. Sister Haward did her duty bravely and kindly while health and strength held out, and has now fallen at her post overborne by the stress of daily toil and struggle pressing too heavily on a weakened nervous system. Patients under her treatment have spoken gratefully and kindly of Sister Haward, and she will long be missed by those who have known her at Simla. The deceased lady was the youngest daughter of the late Mr. Edwin Haward, of Bungay.

### Presentations.

NURSE ELIZABETH THORNTON, of the Nurses' Co-operation, has been presented with a handsome Bible by the inmates of the Wallingford Workhouse Hospital, where she has been taking charge during the past three months. The following inscription is written inside: "With grateful remembrance and best wishes for future welfare."

### Everybody's Opinion.

[Correspondence on all subjects is invited, but we cannot in any way be responsible for the opinions expressed by our correspondents. No communication can be entertained if the name and address of the correspondent is not given, as a guarantee of good faith but not necessarily for publication, or unless one side of the paper only is written on.]

### LADIES AND GENTLEWOMEN.

LUCIE HILL writes: I am interested in the article headed "Ladies and Gentlewomen" in our "Nursing Mirror" of last week. I have a decided leaning towards my own sex, but there are times when I feel ashamed of our narrow-mindedness. We talk a great deal about equality of the sexes, and yet women do more to foster inequality than men do. We never hear men talk about gentlemen as we talk about ladies and gentlewomen. What is a gentleman? What is a lady or gentlewoman? I shall be glad if some more able writer than myself will answer these questions. To my mind a true woman is a lady, and a true lady a gentlewoman; and every woman who behaves as such merits the treatment and respect of both. Of course, we have not all had the same opportunities of education, neither has it been our privilege to descend from a "good old stock." But we can do much to make ourselves truly noble in character and life. Some time ago Lady Henry Somerset defined a lady as one who does not make another feel her inferior, if I mistake not, socially, mentally, or physically. I would sum it up in this, one who does not hurt the feelings of another unnecessarily. I have heard and thought more on this subject since I became a nurse, and am sorry to see such narrow-mindedness in our profession. If we wish to make our nurses true women let us treat them as gentlewomen.

"X Y Z" writes: Surely it does not need an exceptional amount of education to understand that the word "gentlewoman" means simply a woman of gentle birth, and that it is not descriptive of the character but the social position of the person to whom it is applied. While quite agreeing with your correspondent that gentlewomen (in common with all other women) should be of "refined and gentle natures, whose minds are pure and holy," I fail to see how the absence of these qualities can affect the fact of their birth, nor how the possession of them can confer social status on women of humbler origin even though they may be coupled with a "college education." Once the words "lady" and "gentlewoman" had the same significance, then the former much ill-treated word was used indiscriminately to describe either birth or character, and now it simply means a person of the feminine gender, being applied alike to the highest lady in the land and to the "laidy" who sweeps a crossing. Let us hope "gentlewoman" will not follow the same downward course. It is ridiculous to complain because a matron in advertising for a "gentlewoman" to fill a certain post uses the word in its generally accepted meaning and does not recognise a fanciful definition arbitrarily bestowed upon it by uneducated people.

### NURSING IN WALWORTH.

THE HON. TREASURER, Southwark, Newington, and Walworth District Nursing Association, writes: Seeing in your issue of July 30th (July 23rd?) an announcement of a new scheme for district nursing in Walworth, I think your readers may be under a wrong impression, and gather that no nursing is being done there at the present time. I am, therefore, venturing to draw your attention to the tenth annual report of our Walworth District Nursing Association, which I enclose with this. You will see that our association is affiliated to the Queen Victoria Jubilee Institute, and we train two probationers for the institute. Miss L. Ward, our superintendent—whose name may be known to you—recently received the silver medal for efficient service. The home is supported by the Fishmongers' Company, who give £105 a year for a nurse to work amongst the people resident on their Walworth property. It is called the "Benson Home," in memory of the late Miss Eleanor Benson, whose friends raised a fund after her death and gave it to us for the benefit of the sick poor. We undertake to nurse the whole Union of St. Saviour's, which includes all Walworth and Newington, and we are just increasing our staff from five to six nurses. So far we have not had more claims for nursing than we have been able to meet. Our last annual meeting, held at Bishops' House, Kennington, was very well attended. You will see from this, I think, that nursing is being done in Walworth, and I am sure you will agree with us in feeling that it is better in a district that all who care to secure good nursing for the people should unite and support one central home under a competent superintendent. This is a very poor neighbourhood, and our associationhas a great struggle to keep sufficient funds together, and we cannot afford to run any risk of losing our supporters; and I am afraid there are not enough people interested in this district to adequately support two homes. If you are good enough to correct the error, to which I hear the paragraph in your paper has given rise, we should be very grateful. I am writing for our secretary, Miss Gregory, who is away from home. I hope that you will forgive me for troubling you with such a long letter, and that you will ask me any questions about our association if I have not made its objects and methods clear to you. I might add that I have seen Mrs. Bailey and asked her to unite with us, but she cannot see her way to do so.

### A Charming Holiday Resort.

(FROM A CORRESPONDENT.)

HAVING found a delightful mountain nook, I should like to tell others about it, who, like myself, wish for quiet and repose, combined with pure mountain air and lovely scenery. All these advantages are to be met with at La Comballaz Ormonts Vaud, which has been renowned for many years as a health resort, and where comfortable accommodation and good plain food can be obtained at the Hôtel de la Couronne. The terms are very reasonable, from five francs per day (about 4s. 2d.), so that economy as well as health is considered. The altitude is 4,476 feet, and Le Dent du Midi, Les Diablerets, and Mont Blanc are within view. There are plenty of walks and excursions, suitable for the delicate and strong alike. This is really an ideal place for those who like to be away from "the busy hum of men," and yet to be within reach of a daily post, telegraph, and telephone. The tonic properties of the air are decided, and specially beneficial for those suffering from nerve prostration, anæmia, or any chest delicacy. The roads dry up remarkably quickly after rain, and there is no dampness after sunset, as is so often the case in mountain resorts; the sun is hot during the day, but there is always a fresh breeze. August is delightful here, but September is considered the best month. There is an English chaplain here, and the Roman Catholics are also provided for. Travellers from England should go to Lausanne, thence by steamer or rail to Aigle, whence the diligence or carriage would convey them here in three hours. The proprietor is very obliging and speaks English.

### Minor Appointments.

INFECTIOUS DISEASE HOSPITAL, DARWEN.—Miss Emily Erskine was appointed Matron of this hospital on the 4th inst. She was trained ten years ago at the Edinburgh Children's Hospital and the Leicester Infirmary, and since then has been engaged in private and district nursing. She has worked in that capacity for the past three and a half years at Darwen.

WEST HAM UNION WORKHOUSE INFIRMARY.—Miss Margaret Baxter Clark, who was trained at the Aberdeen Royal Infirmary, was appointed Superintendent Nurse at this infirmary on the 11th inst. Miss Clark is at present superintendent of nurses at the East Poor House, Nelson Street, Aberdeen.

THE INFECTIOUS HOSPITAL, KEYCOL HILL, SITTINGBOURNE.—On August 12th, Miss Edith Morris was appointed Matron of this institution. She was trained under the Metropolitan Asylums Board, and has held appointments in the Board's Isolation Hospital at Willesden.

BILLERICAY UNION WORKHOUSE INFIRMARY. — Miss Elizabeth Allen, who was trained at the Stafford General Infirmary, has been appointed Superintendent Nurse of this infirmary. She has held appointments in Stroud Union Infirmary.

### For Reading to the Sick.

"TAKE my yoke upon you and learn of Me, for I am meek and lowly of heart."—*Matt.* xi. 29.

### Verses.

It would be hard with thee if heaven were shut
To such as have not learning! Nay, nay, nay!
He condescends to them of low estate;
To such as are despised He cometh down,
Stands at the door, and knocks. —*J. Ingelow.*

Ye who build the churches of the Lord,
See that ye make the western portals low!
Let no one enter who disdains to bow!
High truths, profanely gazed at, unadored,
Will be abused at first—at last abhorred;
And many a learned, many a lofty brow,
Hath rested, pillowed on a humbler vow
Than keen logicians notice or record—
O, stainless peace of blest humility!
—*Aubrey de Vere.*

If rightly trained and bred,
Humanity is humble, finds no spot
Which her heaven-guided feet refuse to tread. . . .
Love, as Nature loves, the lonely poor!
Search for their worth—some gentle heart wrong-proof,
Meek, patient, kind, and were its trials fewer,
Belike, less happy. Stand no more aloof!
—*Wordsworth.*

Jesus, Who deem'dst it not unmeet
To wash Thine own disciples' feet,
Though Thou wert Lord of All;
Teach me whereby this wisdom meek,
That they who self-abasement seek
Alone shall fear no fall. —*Faber.*

If that in sight of God is great,
Which counts itself for small,
We by that law Humility
The chiefest Grace must call;
Which being such, not knows itself
To be a Grace at all. —*Trench.*

### Reading.

A humble life is a life of prayer. A life of prayer must be a life of humility. If we want to be humble we must pray, and the more we pray the more humble we shall become. And this praying, of course, will not be only praying about humility, but about all sorts of other things, about everything in fact, and especially about very little things. It is most important that we should pray about very little things, not only because if we pray about little things we are sure also to pray about great things, but because every time we pray we acknowledge that we cannot do right of our own accord but that we want God's help, and not only are our prayers answered and the help given so that we may do the little things well for Christ's sake, and also at the same time we learn to be humble, and so come nearer to Him, who humbled Himself for our sake and for our salvation . . . and this would be obeying the command that our Lord has given us to "pray without ceasing."

Of course this does not mean that we are to spend our life upon our knees, or that we are to make any sign that other people could see that we are praying. It means that we continually take about with us the consciousness of the presence of God, and whenever the heart difficulty occurs, and we do not know what to think or decide, or say or do, a thought of prayer goes straight up to Him, and the grace comes that we need.—*H. W.*

### Notes and Queries.

**The contents of the Editor's Letter-box have now reached such unwieldy proportions that it has become necessary to establish a hard and fast rule regarding Answers to Correspondents. In future, all questions requiring replies will continue to be answered in this column without any fee. If an answer is required by letter, a fee of half-a-crown must be enclosed with the note containing the enquiry. We are always pleased to help our numerous correspondents to the fullest extent, and we can trust them to sympathise in the overwhelming amount of writing which makes the new rules a necessity.**

**Every communication must be accompanied by the writer's name and address, otherwise it will receive no attention.**

*Disinfectant.*

(189) Will you kindly tell me of a cheap disinfectant and deodoriser which would do away with the smell from a sink down which the housemaid's pail is emptied? We find permanganate of potash does not answer the purpose.—*D. H.*

Keep the sink absolutely clean with soda, hot water, and a scrubbing brush. Then use Calvert's carbolic acid, No. 5. If the smell persists, the drain is out of order, and must be properly trapped.

*Home for the Aged.*

(190) Can you tell me of any institution or home that would take an old woman, formerly a servant, who is quite childish. She is able to pay a small sum weekly, and therefore is not allowed to be kept in the workhouse infirmary.—*A. M. S.*

The National Society for Promoting the Welfare of the Feeble-minded, 49, Victoria Street, Westminster, would probably be able to help you.

*A Year's Training.*

(191) Please tell me one or two good hospitals that give certificate with year's training. I am going in for monthly nursing after.—*A. K.*

See our advertisement columns or consult "Burdett's Official Nursing Directory" (5s. net, Scientific Press, London).

*Probationer at Guy's.*

(192) I am anxious to become a probationer at Guy's, but I am only 22. Would it be worth while sending in my name now for a vacancy occurring after my twenty-third birthday?—*A. D.*

You should apply direct to the matron.

*Milk Sterilizer.*

(193) I shall be greatly obliged if you will tell me if you know of a reliable make of milk sterilizer suitable for hospital use. One to hold five gallons would be large enough.—*J. A.*

The one devised by Dr. Aymard, of Ipswich, is very good. It is made by G. Bennett, Ipswich.

*An Act of Insubordination.*

(194) I was appointed assistant nurse at an infirmary, after having given the guardians distinctly to understand that I was not competent to undertake the care of imbeciles. One night last month I was told that I must take night duty in the imbecile wards, where there were 23 patients. I declined, as I did not feel competent to do so, and I have been dismissed. Will this disqualify me from taking another appointment under the Poor Law?—*Anxious.*

It will not disqualify you; but unless you obtain a testimonial from the guardians stating the act of insubordination for which you were dismissed, it will probably interfere with your promotion. Why did you not obey orders, and afterwards submit a protest or send in your resignation? You would not be held responsible in such circumstances for anything beyond your knowledge or powers. Loyal obedience to orders is, however, the first qualification of a good nurse.

*Homes for Inebriates.*

(195) Will you kindly tell me any addresses you may know of homes for inebriates?—*M. E. B.*

See page 912, "Burdett's Hospitals and Charities." There is one for women, The Royal Victoria Home, at Bristol.

*Uniform.*

(196) I was a Red Cross Sister in the late Turco-Greek War. I am going for five weeks' holiday, and my people wish me to wear my uniform. (1) Can I? (2) Am I entitled also to wear the Red Cross, which I have worked in my nurse's cloak, or is it considered not the thing to do after the war is over?—*E. H.*

(1) It is not considered good taste on a nurse's part to wear uniform when off duty. There are many excellent reasons for this, but we lack space to give them. (2) It depends upon the conditions under which it was given.

*Short Time Certificates.*

(197) 1. Kindly tell me the names of one or two good hospitals that give certificate for 18 months or two years to paid probationers. 2. Can sufficient experience be gained in a children's hospital for private nursing.—*A. K.*

See "Burdett's Official Nursing Directory" (price 5s., from Scientific Press, London) for list of schools and conditions of training. (2) All authorities on the subject consider three years' training in a general, or in a general and a special hospital, the minimum qualification, and we cannot recommend less or other.

*A Nurse for Canada.*

(198) Is there any society or association that sends nurses abroad? If so, would a nurse with a three years' certificate be likely to be received? I am anxious to go out, preferably to Canada, but do not know where to apply.—*An Inquirer.*

The secretary, the Colonial Nursing Association, the Imperial Institute, W., could possibly help you. And why not apply to the secretary of the Victorian Order of Nurses, founded last year by Lady Aberdeen?

